# Dr. Shields’ Case of Consumption

**Published:** 1852-03

**Authors:** 


### DR. SHIELDS' CASE OF CONSTIPATION.

We have just received a letter from Dr. Shields, communicating the result of the case reported in our last number. He says:

The young lady whose case I reported for your Journal has returned home with her health perfectly restored. My report closed with her departure from New Albany on a visit to her friends in a neighboring city. After she left home, she took exercise as she was able to bear it, and omitted all medicine. Not many days after she was gratified by the return of her catamenia, and has since that event enjoyed better health than she had done at any time since the first appearance of her menses.

New Albany, Feb. 10th, 1852.

